# The aftermath of asbestos prohibition in industry and its association with malignant mesothelioma in the south of Iran: An enduring predicament yet to be resolved

**DOI:** 10.1002/hsr2.70117

**Published:** 2024-10-06

**Authors:** Alireza Rezvani, Reza Shahriarirad, Sahar Jahanshahi, Damoun Fouladi, Maryam Tavallali, Bizhan Ziaian, Mohammad Javad Fallahi

**Affiliations:** ^1^ Thoracic and Vascular Surgery Research Center Shiraz University of Medical Science Shiraz Iran; ^2^ Bone Marrow Transplantation Center, Nemazi Hospital Shiraz University of Medical Sciences Shiraz Iran; ^3^ Student Research Committee Shiraz University of Medical Sciences Shiraz Iran; ^4^ College of Agricultural Engineering, Department of Water Engineering Shiraz University Shiraz Iran; ^5^ Department of Internal Medicine, Nemazee Hospital Shiraz University of Medical Sciences Shiraz Iran

**Keywords:** asbestos, mesothelioma, occupations

## Abstract

**Purpose:**

Malignant Mesothelioma (MM) is a rare malignancy of the serosa membranes with a high mortality rate and long latent period. The relationship between a group of mineral fibers known as asbestos and mesothelioma is now well accepted in which people can be exposed to these fibers by various means during their lifetime and has been its usage has banned in many countries, such as Iran, which announced its gradual elimination from 1999 over a period of 7 years by using safe substitutes. However, the mineral particles are able to sustain itself in the environment, air, water, and soil and on the other hand, symptoms may take up to half a century to develop in exposed individuals. Also, there remains a shortage of comprehensive investigation on the effects of asbestos exposure within the familial context (household or domestic exposure) or on individuals residing in proximity to asbestos mines or factories (environmental exposure). Based on the high number of MM cases in Iran, and also our hypothesis that residuals of asbestos in the environment and petroleum products may be the etiological factor for MM, we conducted this study to evaluate the clinic epidemiological features of MM in the south of Iran its relation to possible asbestos exposure.

**Methods:**

In this study, we analyzed the demographic features and occupations of confirmed cases of MM in Shiraz, southern Iran along with the follow‐up of the patients’ disease from 2008 to 2018, while also comparing the features of our patients with a control group compromising of 105 non‐MM patients.

**Results:**

Among the 35 confirmed cases of MM, with an average age of 61 years, 9 (25.7%) were female, and 26 (74.3%) were male. During our assessment, 12 patients had already died, with a mean time of 11.26 months post‐diagnosis. Our findings revealed a higher prevalence of MM among housekeepers and employees of oil companies. In comparison to the control group, individuals with occupational exposure and those residing near refinery locations were at a heightened risk of developing MM. However, based on regression analysis, only occupations associated with refineries exhibited a significant correlation with MM (*p* = 0.028; OR: 14.602; 95% CI: 1.328–160.499).

**Conclusion:**

Both occupational and para‐occupational exposure demonstrated a significant correlation with MM, whereas our regression analysis did not affirm geographical and environmental factors as contributors to MM. Despite the industry's prohibition of direct asbestos usage, the persistent existence of asbestos particles in the environment for decades, coupled with the long latency period of MM, warrants further investigation. Health authorities and policymakers should recognize this potential hazard, prompting an enhancement of early detection within at‐risk groups.

## 1 INTRODUCTION

Malignant Mesothelioma (MM) is a rare and lethal malignancy that affects the serosal membranes, including the pleura, peritoneum, pericardium, and tunica vaginalis. The typical survival rate for MM is 4–18 months post‐diagnosis, with a discouraging 10% 5‐year survival rate.^1^ This cancer significantly contributes to the escalating mortality rates observed in countries such as the United States, the United Kingdom, and Japan.^2^ Malignant pleural mesothelioma is the most common form of MM, with a median survival rate of 1 year and a higher incidence in men than women, believed to be due to men's occupational exposure to potential risk factors.^3^, ^4^ Numerous factors contributing to the development of MM have been identified, such as environmental exposures, genetic predisposition, viral contamination, and radiation. However, the challenge in thoroughly assessing the impact of these risk factors and their subsequent molecular effects arises from the delayed diagnosis of late‐stage MM and the extended latency period between certain exposures and the actual diagnosis. Among the factors causing MM, asbestos exposure is known to be the primary and most significant cause.^5^

Asbestos, a group of six minerals, maintains its enduring impact on the industry due to its fire‐resistant qualities, leading to widespread use in manufacturing sectors like shipbuilding and insulation supplies.^6^ Moreover, as a common material in construction, it poses a significant health risk due to its degrading over time, releasing fibers into the air during demolition and remodeling activities.^7^, ^8^ These tiny threads, in contrast to bigger particles, are challenging for the body's defense mechanisms to remove, leading to a cascade of pathophysiological events that may result in chronic inflammation and mesothelioma cancer.^9^

Asbestos fibers present in various environments raise safety concerns, particularly in landfills, where they can remain suspended in the air for up to 72 h before affecting soil and water supplies.^10^ Inhalation of asbestos activates proto‐oncogenes, causing DNA damage and inflammation, ultimately leading to tumor formation and the risk of aggressive cancer, particularly in the chest and abdominal cavities.^9^ Asbestos fibers can permeate the mucosa of animals’ stomachs and intestines, which highlights the significance of examining the distribution of asbestos fibers in the environment.^11^, ^12^ Previous discoveries of asbestos fibers in beer raise concerns, especially given the presence of fibers measuring 0.5 nm in length and 0.01 nm in diameter. These dimensions suggest that such material could potentially pass through filtering systems and enter city drinking water supplies.^13^ The detrimental effects of asbestos exposure at work are evident, with over 70,000 workers losing their lives in 2019, constituting a major health concern as asbestos is linked to 78% of occupational malignancies in Europe.^14^

Despite significant advancements in risk mitigation, asbestos has been prohibited for more than 50 years in many industrialized and developing countries.^15^ While the bans on asbestos are a positive step, more action is needed to address its continued threat to global health due to its carcinogenic characteristics.^16^ Despite the prohibition of asbestos production, import, and use in numerous regions, the peak frequency of MM remains unreported. This lack of reporting is attributed to the latency period between asbestos exposure and the occurrence of MM, which typically manifests 30‐50 years after initial exposure. On the contrary, in newly industrializing countries in Asia, Eastern Europe, and South America, where asbestos use is increasing, the incidence of MM is currently relatively low. However, it is worth noting that underreporting of mortality and a lack of incidence data have been observed in many countries. Anticipated to increase in the upcoming years, the incidence of MM faces challenges due to the prolonged latency period and the fact that not all exposed individuals manifest the disease. This underscores the importance of continued vigilance and research to fully comprehend the environmental effects of asbestosis leading to MM.^17^, ^18^

While numerous studies have delved into the repercussions of occupational asbestos exposure within cohorts of workers, there remains a shortage of comprehensive investigation on the effects of asbestos exposure within the familial context (household or domestic exposure) or on individuals residing in proximity to asbestos mines or factories (environmental exposure). Our objective in this study was to assess the impact of asbestos of MM among three distinct groups; workers employed at the southern Iran factories (experiencing occupational exposure); their cohabitants (familial exposure resulting from fibers on the workers’ clothes or hair); and individuals living in southern Iran, or nearby towns (experiencing environmental exposure due to outdoor pollution associated with the factory). Living near a refinery was considered residential houses located in the factories and occupants living in the organizational and institutional houses in the refinery and petrochemical companies (the maximum distance is five kilometers). We aimed to assess the clinic epidemiological aspects of MM and its potential relation to asbestos exposure years after the initial exposure. We also aimed to emphasize the urgent need for action regarding the consequences of asbestos, even decades after its ban.

## 2 MATERIALS AND METHODS

This retrospective study was conducted at Namazi Hospital, affiliated with Shiraz University of Medical Sciences, in Shiraz, Fars Province, Southern Iran, spanning from 2008 to 2018. Hospital records were meticulously scrutinized to identify all cases diagnosed with MM and confirmed through Immunohistochemistry and relative guidelines.^19^ In the hospital records, files for 47 patients with an MM diagnosis were identified. Subsequently, the hospital records were evaluated and the individuals were contacted through a phone call and asked a series of questions to obtain their demographic data and information regarding environmental exposures. The date of the last follow‐up was till the end of 2022.

Among the identified cases, 12 were excluded from the study due to the unavailability of contact information in their files or a lack of response after attempted communication. Additionally, in instances where the patient had deceased, relevant information was sourced from close relatives. Variables such as sex, age, previous occupation (based on the International Standard Classification of Occupations (ISCO‐08) classification), living area geographies, the time of MM diagnosis, history of smoking, and familial history of MM were collected. In parallel, a control group comprising 105 patients were selected from other departments and hospitals with a similar affiliation and location to our case group and mainly consisted of patients with traumatic and orthopedic injuries. The hospitals were chosen based on their similar catchment areas to minimize selection bias and ensure demographic and clinical similarity. This selection was made to ensure a comparable distribution in terms of age and gender for subsequent comparison.

### 2.1 Statistical analysis

Statistical analyses were performed using the statistical package for social sciences (SPSS Inc., Chicago, Illinois, USA) version 26.0, employing the Chi‐square/Fisher's exact test to compare the relationship between descriptive variables and MM. Data were presented as mean ± SD, and proportions were used as suitable. To assess the association between the variables and MM, odds ratios (OR) were calculated. Furthermore, logistic regression analysis was performed to evaluate potential risk factors for MM in our study. A p‐value of less than 0.05 was considered significant.

## 3 RESULTS

### 3.1 Overall

Among the 35 MM cases, 9 (25.7%) were female, and 26 (74.3%) were male. The mean age of the patients was 61.05 (SD = 15.33) years, with the youngest case being a 27‐year‐old woman and the oldest an 86‐year‐old man. The median latency period of the cases in our study was 10 months [Q1–Q3: 4–12; range: 1–48]. Table 1 demonstrates the features of the patients in our study. As demonstrated, there was no significant difference between the case and control group regarding their gender, age, and smoking status (*p* > 0.05).

**Table 1:** Baseline demographical and social features of patients included in our study, based on the diagnosis of mesothelioma in Southern Iran.

Variable		Total; *N* = *140*	Malignant Mesothelioma (%) *n* = *35*	Control Group (%) *n* = *105*	*P*‐value^a^
Gender; *n* (%)	Male	104 (74.3)	26 (74.3)	787 (74.3)	1.00
	Female	36 (25.7)	9 (25.7)	27 (25.7)	
Age (years); mean ± standard deviation		61.1 ± 15.4	61.1 ± 15.3	61.1 ± 15.5	0.98
Age group (years); *n* (%)	≤40	20 (14.3)	5 (14.3)	15 (14.3)	1.00
	41–50	10 (7.1)	3 (8.6)	7 (6.7)	
	51–60	25 (17.9)	6 (17.1)	19 (18.1)	
	61–70	46 (32.9)	11 (31.4)	35 (33.3)	
	>70	39 (27.9)	10 (28.6)	29 (27.6)	
Smoker; *n* (%)	Positive	56 (40.0)	16 (45.7)	40 (38.1)	0.43
	Negative	84 (60.0)	19 (54.3)	65 (61.9)	
Family History; *n* (%)	Positive	0 (0)	0 (0)	0(0)	‐
	Negative	140 (100)	35 (100)	105 (100)	
Living Location Near Refinery; *n* (%)	Positive	27 (19.3)	13 (37.1)	14 (13.3)	**0.002**
	Negative	113 (80.7)	22 (62.9)	91 (86.7)	
Occupational Exposure to asbestos; *n* (%)	Positive	7 (5.0)	6 (17.1)	1 (1.0)	**<0.001**
	Negative	133 (85.0)	29 (82.9)	104 (99.0)	
Mortality; *n* (%)	Positive	23 (16.4)	23 (65.7)	0(0)	**<0.001**
	Negative	117 (83.6)	12 (34.3)	105 (100)	

*Note*: Bold values indicate significant association.

a P‐values are calculated based on the Chi‐Square/Fisher's Exact test or the independent sample t‐test.

**Figure 1:**
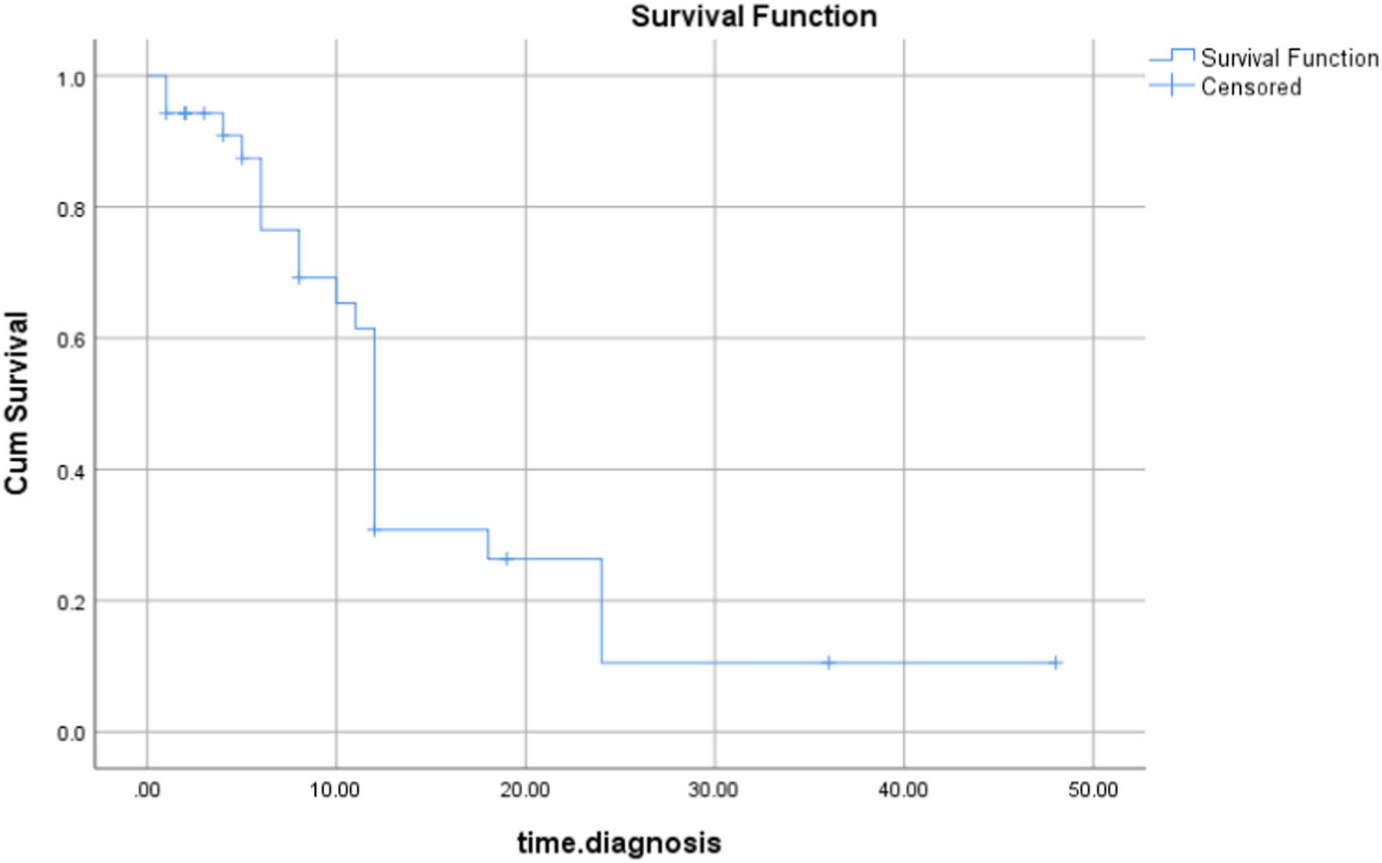
The Kaplan–Meier curve among patients diagnosed with Malignant Mesothelioma, indicating a decrease in the chance of survival over the years. (x axes refers to “months since diagnosis”)

At the time of the study, 34% of cases (12 patients) had died, and 66% (23 patients) were still alive. Figure 1 demonstrates the Kaplan‐Mayer analysis of the mortality in our study. The median survival time was 12 months (IQR: 16; 95%CI: 11.42–12.57). The survival probability (95%CI) for our MM patients at 3 months was 94.3% (86.7–100%), at 6 months was 76.5% (61.0–92.0%), at 12 months 30.8% (13.2–48.4%) and beyond 12 months was 26.4% (9.4–43.5%; average 18 months). There was no significant difference between the latency period and mortality in our study (*p* = 0.25).

### 3.2 Demographic and social features

The highest frequency of the disease (31.4%) was in the age group of 61 to 70 years, and the lowest (8.6%) frequency was among the 41 to 50 years age group. Familial history of MM was negative in all patients in our study. Among the patients in our study, 16 (46%) were either active or passive smokers, while 19 (56%) were non‐smokers. Statistical analysis showed no significant association between smoking and the prevalence of MM (*p* = 0.433). Our results demonstrated a significant association between the prevalence of MM and the patients’ occupation (*p* = 0.006) (Table 2).

**Table 2:** Distribution of various occupations based on patients with and without malignant in southern Iran.

Variable	Malignant Mesothelioma (%) *n* = *35*	Control Group (%) *n* = *105*	OR (95%CI); *P*‐value^b^	
			Univariate	Multivariate
Occupational Exposure				
Oil company	6 (17.1)	1 (1.0)	−0.06	−0.22
Nonoccupational (Environmental/Domestic)				
Construction	4 (11.4)	12 (11.4)	18.00 (1.63–198.51); **0.02**	14.13 (0.23–215.89); 0.06
Farmer	3 (8.6)	17 (16.2)	34 (2.94–392.85); **0.005**	22.89 (1.60–328.1); **0.02**
Fisherman/woman	2 (5.7)	1 (1.0)	3.00 (0.12–73.64); 0.50	3.34 (0.12–95.71); 0.48
Carpet‐weaver	2 (5.7)	2 (1.9)	6.00 (0.34–107.4); 0.22	4.82 (0.23–101.30); 0.31
Housekeeper	8 (22.9)	19 (18.1)	14.25 (1.47–138.27); **0.02**	4.54 (0.15–137.83); 0.38
Driver	1 (2.9)	7 (87.5)	42.00 (2.14–825.72); **0.01**	31.71 (1.23–820.20); **0.04**
Teacher	1 (2.9)	10 (9.5)	60.00 (3.14–1147.29); **0.01**	36.63 (1.62–830.3); **0.02**
Others^a^	8 (22.6)	36 (26.6)	27.00 (2.84–256.52); **0.004**	18.72 (1.52–229.95); **0.02**

*Note*: Bold values indicate significant association.

Abbreviations: CI, Confidence interval; OR, Odds ratio.

a Other occupations included physician, sales manager, electronic industries employee, university student, a food company employee, water laboratory technician, retired army member, photographer, and mattress manufacturing factory employee.

b Values are compared to “Oil company” (Occupational) as indicator. Bold values indicate significant association.

Occupations and living locations of the participants were categorized based on their relation to a refinery or a living location near a refinery and were compared between the case and control groups. In the case group, 6 individuals (17.1%) were employed by oil companies, whereas in the control group, only 1 person (1%) worked for an oil company. A significant association was observed between the occupation of oil company employees and MM (OR (95%CI): 21.51 (2.49–185.95); *p* = 0.001). Furthermore, exposure and contact with asbestos based on the geographical living location also demonstrated a significant association with the prevalence of MM. (Living location near refinery: Total: 27 (19.3); Case: 13 (48.1%) versus Control: 14 (51.9%); OR (95%CI): 3.84 (1.58–9.33); *P*‐value: 0.005).

We evaluated our data, including age, gender, smoking status, living location in relation to refinery, and occupation based on logistic regression analysis to evaluate risk factors for MM in our study. Based on our model, occupational exposure versus nonoccupational exposure showed a significant correlation with MM (*p* = 0.03; OR: 14.60; 95%CI: 1.33–160.50).

## 4 DISCUSSION

Asbestos has been discovered and mined by mankind for thousands of years.^20^ The modern asbestos industry started in the 1800s and the production and use have raised in the following years.^21^ The link between occupational exposure to asbestos and mesothelioma was first proposed in 1935.^22^ The popularity comes from the unusual and practical properties of theses fibers such as heat resistance, flexibility and high tensile strength, thermal, electrical, and sound insulation.^23^, ^24^

Followed by the extensive use of asbestos in the industry, the annular mortality due to MM increased dramatically in the 1900s.^25^ That was a wakeup call for many countries to start banning and limiting the use of these hazardous fibers in industry. Laws on asbestos ban started with Denmark in 1972 with the ban of thermal and noise insulation and waterproofing use of asbestos and soon after, other countries came up with new legislation limiting the use of asbestos too; Iceland was the first country to completely ban asbestos use in 1983.^26^ The effect of the new legislation can be seen from 1980 when worldwide asbestos usage started to decline. By 2010, 52 countries banned all forms of asbestos use; but Iran was still one of the 8 countries (Brazil, China, India, Iran, Kazakhstan, Russia, Thailand, and Ukraine) with the highest usage in 2003.^21^, ^27^ With the estimated median latent period of 32 years after the first occupational exposure, it is obvious that MM is still going to be of a concern in the following decades and it can be expected that the peak has not yet reached in Iran like other countries such as Italy which banned asbestos in 1992.^25^, ^28^, ^29^ Additionally, it is not anticipated that the incidence of MM will decline in the upcoming years.^30^ Due to incomplete asbestos bans in certain regions, asbestos in still‐existing buildings, the market's continuance of asbestos‐containing items, and international trade, which includes asbestos imports, are all ongoing challenges related to asbestos. As such, it is critical to give continued research efforts focused on understanding and reducing the long‐term effects of asbestos usage the greatest importance.

Our first focus was the occupational exposure. In our study, oil company workers constituted the second most frequent group, following housekeepers, who were diagnosed with MM. A study on occupational exposure in 2002, involving 1445 confirmed cases of mesothelioma in the USA, revealed that shipbuilding, the US navy, and the construction industry were the top three industries with the highest incidence of MM, while the oil and chemical industry ranked fifth. Considering that the oil and gas industry is the predominant sector in the south of Iran, it is reasonable to assert that our results are comparable to those of the mentioned study.^31^ Another study on employees in the refinery and petrochemical sector in Lambton County, Ontario, also confirmed that employment as a maintenance worker in this industry was associated with an increased risk of asbestos‐related MM.^32^ In a similar study on 272 cases of MM in Iran from 2006 to 2010, which was reported by the ministry of health of Iran, high‐risk occupations included construction workers with 20% of cases followed by oil company workers with 13.3%.^33^ Our regression analysis revealed a significant correlation between MM and occupational exposure, one of the three aspects under investigation based on our initial hypothesis. These results align with existing literature, affirming the well‐established fact that occupational exposure to asbestos correlates with MM occurrence.^34^, ^35^ Workplace safety, psychosocial support, and education are vital, particularly for vulnerable populations facing disparities.^36^, ^37^ The study advocates for stricter safety measures and increased awareness. Many individuals may not fully grasp the potential risks tied to asbestos exposure, causing them to neglect regular health check‐ups. Nonetheless, grasping the prognosis becomes essential, as it not only offers valuable insights into the progression of the disease but also plays a pivotal role in guiding treatment decisions and healthcare choices.^38^

Aside from occupational exposure, consideration should be given to other forms of exposures, including para‐occupational exposure within the familial context, including household or domestic exposure, and also environmental exposures. While the risk associated with these exposures may be lower compared to occupational exposure, it is imperative to recognize that numerous individuals, particularly women, are potentially at risk through various means throughout their lifetime.^31^, ^39^, ^40^ Recent studies have confirmed that the risk of MM increases with cumulative asbestos exposure.^41^ In 2011 Alessandro Marinaccio and his colleagues studied on MM epidemic which demonstrates occupational asbestos exposure was in 69.3% of MM cases (*N* = 4577 cases), while 4.4% was due to cohabitation with someone (generally, the husband) who was occupationally exposed, 4.7% by environmental exposure from living near a contamination source and 1.6% during leisure activity.^42^ Excluding the 6 oil company employees, 7 of our patients lived near the oil company which could be a potentially contaminated source which our study showed that it increases developing MM by threefold. Although our regression model analysis did not prove environmental exposure to be a relative risk factor, the most frequent occupation with MM were housewives, strengthening the initial hypothesis that domestic and familial expose can increase the risk of MM. Para‐occupational exposure, also known as household exposure, involves asbestos‐exposed workers acting as vectors, transporting fibers to their homes. This exposure pathway, sometimes termed household contact or take‐home exposure, specifically pertains to indirect asbestos exposure from high‐risk occupational settings to households or other environments where individuals, typically family members, interact with the worker. Unlike residential exposure pathways involving asbestos‐contaminated insulation or soils, para‐occupational exposure is closely linked to the occupational setting.^43^ Common activities contributing to para‐occupational exposure include laundering contaminated work clothes, with simulation studies suggesting potential take‐home exposures to household contacts as a fraction, possibly 1%, of occupational exposures.^44^ Despite limited data on exposure concentrations, lung tissue asbestos burden among women para‐occupationally exposed to mesothelioma demonstrated similarities to men with moderate occupational exposure.^31^ Besides laundering, other activities such as cleaning and the use of the worker's vehicle can contribute to para‐occupational exposure. Understanding this route of exposure is crucial, varying significantly based on the occupational source.^43^ Numerous reported mesothelioma cases among family members of workers in asbestos‐exposed industries highlight the relevance of para‐occupational exposure, supporting the notion that healthcare workers and policymakers should be attentive to potential correlations in this regard.^43^, ^45^, ^46^, ^47^, ^48^

Numerous studies have been conducted worldwide to raise awareness around asbestos carcinogenicity which led to this strict legislation we have today. Regarding our study, mean age of the patients was 61 years and male to female ratio was 3 to 1 which is corresponding with many articles such as Haining Yang et al. that demonstrated most of the patients are between 50 and 70 years of age and that men are more at risk due to occupational exposure than women.^3^ Furthermore, their study, similar to ours, stated that there is no evidence showing an association between MM and smoking. Contrary to our study, Abdel‐Hamid MA et al. regarding the environmental and occupational risk factors among malignant pleural mesothelioma, reported that smoking was considered as a risk factor for MM of pleura.^49^

Due to the tumor's aggressive nature and limited efficacy of current therapies, the median survival of patients with MM from the time of diagnosis is about 12 months, which is consistent with our findings demonstrating an average of 11 months.^24^, ^39^ The Kaplan‐Meier curve highlights the aggressive nature of MM by showing a decreasing survival rate over time. Given that none of the patients had a positive family history, environmental factors appear to have a greater influence than genetic propensity.^35^ Moreover, recognizing the extended latency period of asbestos‐related illnesses, the discussion extends to the challenges in health monitoring, access to specialized care, and financial obstacles. To counter these challenges, the automotive and textile industries have adopted safer materials like ceramic and synthetic fibers, respectively, reducing health risks. Green construction methods incorporate sustainable alternatives such as natural clay and recycled glass.

We initially documented 47 patients with MM over a span of 10 years at our study location. Compared to earlier studies in Iran, the prevalence of pleural MM appears to be on the rise. Several studies have been reported in Tehran, which demonstrated 40 cases of pleural MM between 1996 and 2008,^50^ 66 cases between 2001 and 2008,^51^ and a final report based on the lung cancer registry documented 60 cases (10%) of pleural MM among 600 lung cancer patients from 2010 to 2013, with 36 of these patients (61.7%) having a history of asbestos exposure for an average of 36.1 years.^52^ These evidence suggest an increasing incidence of the disease or improvements in diagnostic methods.

Our study has limitations, such as the lack of a detailed environmental assessment of asbestos particles and the specific geographical radius under influence by refineries. Furthermore, clinical features and long‐term survival of patients, as well as the changes in the latency period and duration of exposure were not documented as they were not the focus of our study; However, conducting further long‐term follow‐up and multicentral studies in nations where asbestos has been used recently can contribute valuable evidence regarding this hazardous carcinogen.

## 5 CONCLUSION

Occupational and household exposure exhibited a significant correlation with MM, while our regression analysis did not confirm geographical and environmental factors as contributors to MM. However, on one hand, considering that asbestos particles can persist in the environment for decades and, on the other hand, given the long latency period of MM, the environmental and domestic exposure risk in MM remains a matter of debate that necessitates further investigation. Nevertheless, healthcare workers and policymakers should be cognizant of the potential correlations in this regard and provide early detection among potential risk groups.

## AUTHOR CONTRIBUTIONS

Alireza Rezvani: Conceptualization; Investigation; Supervision; Validation; Project administration. Reza Shahriarirad: Conceptualization; Methodology; Formal analysis; Writing—review and editing; Writing—original draft; Data curation. Sahar Jahanshah**i**: Data curation. Damoun Fouladi: Writing—original draft; Writing—review and editing. Maryam Tavallali: Writing—original draft; Writing—review and editing. Bizhan Ziaian: Supervision; Investigation. Mohammad Javad Fallahi: Supervision; Validation; Investigation.

## COMPETING OF INTERESTS STATEMENT

The authors declare no conflicts of interest.

## ETHICS APPROVAL

The study was approved by the Research Ethics Committee of the School of Medicine‐Shiraz University of Medical Sciences (Ethical Code: IR. SUMS. MED. REC.1398.113). Permission to carry out the study and access patient records was sought from the respective university administrators, and the study was conducted in compliance in accordance with the relevant guidelines and regulations and the Declaration of Helsinki and was also approved by the ethics committee of the university.

The lead author, Reza Shahriarirad, affirms that this manuscript is an honest, accurate, and transparent account of the study being reported; that no important aspects of the study have been omitted; and that any discrepancies from the study as planned (and, if relevant, registered) have been explained.

## CONSENT TO PARTICIPATE

Written informed consent for participation was obtained from the patients or their next‐of‐kin.

## FUNDING

This research did not receive any specific grant from funding agencies in the public, commercial, or not‐for‐profit sectors.

## TRANSPARENCY STATEMENT

The lead author Reza Shahriarirad affirms that this manuscript is an honest, accurate, and transparent account of the study being reported; that no important aspects of the study have been omitted; and that any discrepancies from the study as planned (and, if relevant, registered) have been explained.

## Data Availability

The data that support the findings of this study are available from the corresponding author upon reasonable request.
